# Displaying pride: Variation by social context, ethnic heritage, and gender?

**DOI:** 10.1371/journal.pone.0285152

**Published:** 2023-04-28

**Authors:** Hugo Sanchez Hernandez, Arpine Hovasapian, Belinda Campos

**Affiliations:** 1 Department of Psychological Science, University of California Irvine, Irvine, California, United States of America; 2 Department of Chicano/Latino Studies, University of California Irvine, Irvine, California, United States of America; The Hong Kong Polytechnic University, HONG KONG

## Abstract

Pride is universal; however, the complexities linked to its social status functions and implications for social relationships suggest the possibility of variation in its display. Drawing from empirical evidence, this study examined whether displayed pride would vary by social context (i.e., whether the target was a competitor or a loved one), ethnic heritage (i.e., membership in individualistic or collectivistic cultural groups) and by gender. Young adults (N = 145) verbally described a pride experience to an imagined competitor, loved one, stranger or in a no-context control condition. Results showed similarity in displayed pride across the four contexts. However, some ethnic group and gender variations were observed. Latino/a/x Americans displayed less pride verbally than European Americans while women displayed more than men. These findings contribute to a better understanding of how people manage the display of pride and suggest that ethnic and gendered motivations for managing pride displays are relevant to a comprehensive understanding of interpersonal emotion regulation.

## Introduction

Expressing positive emotions is generally important for fostering mutually rewarding interactions [1], but compared to other positive emotions, pride is particularly complex. Pride is a discrete emotion for which much evidence of universality exists [2–4], but its display is highly relevant to social status and does not always elicit socially rewarding responses [5]. For these reasons, managing the display of pride is an important and motivation-laden element of emotional communication. While people recognize the experience and prototype of a pride display, they may be motivated to display pride less in situations in which doing so risks bringing about negative social consequences. This is likely to be most true for people socialized to cultural and gender norms that emphasize attunement to the potential negative social consequences that pride can elicit. In short, both situational and individual factors may influence how and when people display pride. The current study examined three potential sources of variation in how pride is displayed: social context, ethnic heritage, and gender. The study provides insight into the factors that shape how people display pride specifically and interpersonal emotion regulation broadly. Furthermore, the study outlines the theoretical and empirical reasons why people might be motivated to manage their pride experiences.

### Pride

Emotions are inherently social. They help people navigate and respond to social interactions appropriately [6]. The expression of positive emotions specifically facilitates the creation of social bonds and strengthens social relationships [1]. Although pride is a positive emotion, it is distinct from other positive emotions because of its particularly complex social functions. Displays of pride alert members of a social group to one’s achievements and signal that one is worthy of high status [3, 7]. Status is generally considered desirable and may be actively sought but displaying pride can elicit upward comparisons and evoke negative emotions (e.g., envy) in observers [8, 9]. For example, Kalokerinos et al. (2014) showed that participants evaluated winners who expressed more positive emotions more negatively than winners who expressed fewer positive emotions. This possibility of negative social consequences has implications for the motivated management of pride. Pride experience may be universal, and people can recognize what its behavioral display looks like, but there may be variation in how people manage their pride.

### Why expect variation by social context?

There are reasons to expect that pride management may vary by social context. For example, individuals may be motivated to display their pride with their loved ones because they are expected to respond positively and the potential for negative social consequences is low. However, in competitive contexts, the possibility for negative social consequences may be salient and individuals may actively manage how pride is displayed. This prediction is consistent with the self-evaluation maintenance model [10]. According to the self-evaluation maintenance model, if the performance of a high-achieving individual is relevant to an observer (e.g., they competed with the observer for something), the observer’s self-evaluation will suffer when they compare themselves to the high-achieving individual [10]. For this reason, a person may not want to call attention to their achievement to prevent this negative scenario. In support of this possibility, at least one study has found that participants inhibited expressions of pride more towards observers for whom the achievement was relevant [11]. This suggests that people may be especially motivated to inhibit their pride displays in competitive contexts.

### Why expect variation by ethnicity?

Beyond social context, pride displays may also differ according to people’s backgrounds. The pride experience and its nonverbal display are universal and cross-culturally recognized [4, 12–14]. From an evolutionary perspective, pride seems to have adaptive functionality, and there are cross-cultural agreements on what causes pride and how pride leads to perceived status gains [15–18]. However, despite convergent views on pride’s functions, culture still shapes the emotions people *prefer* to feel [19, 20], and may influence the extent to which people are motivated to *display* pride [21]. Different cultures may have their own norms around when to engage in unabashed status-signaling pride displays and these displays might be considered maladaptive in particular contexts. For this reason, people might be motivated to adapt their pride displays accordingly, balancing the benefits and costs of signaling their status gains but also potentially being perceived as “showing off.”

Indeed, researchers have found differences in the extent to which pride is valued in individualistic and collectivistic cultures [22], with members of more collectivistic cultures finding pride to be undesirable. For example, studies have found that individuals of Asian heritage (i.e., Chinese descent) are perceived to display less pride when outperforming another person who shares their ethnic heritage than individuals of European American heritage [23]. However, the collectivistic cultures most often studied are those of East Asia [e.g., 22], and there are likely to be differences in cultures that emphasize different forms of collectivism (e.g., Latin American). East Asian and Latin American cultures are both considered collectivistic, but increasing empirical evidence shows that they differ on various dimensions of positive emotions [e.g., gratitude; 24], including their beliefs around the appropriateness of experiencing and expressing them [19]. Specific to pride, Scollon and colleagues (2004) found that not only did people from Hispanic cultures report higher (not lower) levels of pride than those who were European American, but they also reported much higher levels of pride than those who were from Asian cultures (i.e., Asian American, Japanese, and Indian). Other research also shows variation in pride and positive emotion expressions generally between members of Asian and Hispanic collectivistic cultures [21, 25, 26], demonstrating that the nuanced links between collectivism and pride are worthy of additional empirical investigation. Simply put, there may be more cultural variation in the way that pride is displayed than is currently understood. Due to exposure to cultural norms that emphasize the potential negative social consequences of pride, individuals whose ethnic heritages come from Asian collectivistic cultures may be less motivated to display pride to the same degree as those whose ethnic heritage comes from individualistic cultures (i.e., European American heritage) *and* from Latino [27] collectivistic cultures that emphasize positive emotion expressivity [28]. This nuanced thinking drives this study’s examination of ethnic heritage variation in pride displays.

### Why expect variation by gender?

In addition to culture, there may also be gender variation in how pride displays are managed. Some researchers argue that men experience pride more than women [4] while other studies indicate that women and men experience similar levels of pride [29]. Although the relation of gender with pride experience is not fully delineated, there may be variation in how comfortable women and men are with *displaying* pride. Findings on status-saliency give insight into this potential variation. In competitive contexts, when they know that a social ranking of performance will occur, women do not perform as quickly or as well as men [30]. One explanation for this, as argued by Schram and colleagues (2019), is that status rankings motivate women less than men. This explanation, and considering how important status is in the way women are perceived [31], might suggest that women are more attuned to the possible negative social consequences of overt status-seeking. If so, they may be less motivated to engage in the status-signaling of their achievements (i.e., displaying pride). This possibility drives this study’s examination of gender variation, including the possibility that gender would interact with social context due to the salience of status-signaling in particular contexts (i.e., competitive contexts).

### Current study

This study aims to contribute new insight into the ways that people manage the display of pride, both verbally and nonverbally, across the situations they are in, across ethnic heritages linked with individualistic and collectivistic cultures, and across women and men. To do so, the study employs a behavioral task (i.e., a task that allows researchers to observe participant behavior) that blends various strengths of past influential studies that have used self-report methods (e.g., numerical scales, choosing photograph expressions) or imagined scenarios that evoke emotion to examine pride and positive emotion expressions [8, 11, 23]. Like one of the experiments conducted by van Osch and colleagues (2019), the current study focused on spontaneously capturing how individuals would behaviorally describe a prideful accomplishment that was real to them while in a controlled laboratory environment.

The authors hypothesized that pride would be displayed with less verbal intensity to a competitor than to a loved one, stranger, or control (no-context) condition; that Asian Americans would display pride with less verbal intensity than Latino/a/x Americans and European Americans; and that women would display pride with less verbal intensity than men. Due to mixed findings in past research, the authors did not formulate hypotheses for nonverbal displays; research finds that pride is characterized by recognizable nonverbal behavioral displays (e.g., raising the head, arms raised), but there are discrepant findings on whether all forms of pride are characterized by certain expressions (e.g., Duchenne smile, eye gaze directed straight ahead) [3, 32–34]. In addition, the study also explored whether there were pride display differences between Latino/a/x Americans and European Americans and whether any ethnicity or gender differences varied by social context.

## Method

### Participants

Participants (*N* = 145; 74.5% women; *M*_*age*_ = 21 years) were undergraduate students attending a public university. To test the research questions regarding ethnicity, a sample of 117 participants were drawn from the larger dataset [35]. These participants self-identified as Asian American (50.4%), Latino/a/x American (34.2%), and European American (15.4%). The majority reported that they had been born in the United States (72.6%), that they came from families that speak languages other than English at home (91.5%), and that their families were lower middle class (53%; there were also 19.7% upper working class; 18.8% upper middle class; 7.7% lower working class; 0.9% upper class). Procedures were approved by the University’s Institutional Review Board.

### Procedure

Participants were recruited through the university participant pool. Upon arriving at the lab, participants gave written informed consent and were led through study procedures.

#### Emotion expression task

Participants were seated at a table in front of a dome ceiling camera and were instructed, via intercom, to talk to someone they imagined was sitting across from them about a school-related accomplishment that evoked personal pride. The participants were randomly assigned to one of four social context conditions. For the “competitor” condition (*n* = 37), they imagined talking to a competitor (i.e., someone from class that the participant knows but is not very close with, someone they compete with, or someone they want to do better than) who initially believed they would outperform the participant but did not. For the “loved one” condition (*n* = 39), they imagined talking to a significant other, family member, or close friend. For the “stranger” condition (*n* = 34), they imagined talking to someone they had just met. Those in the “no-context” control condition (*n* = 35) also talked about a school-related accomplishment that evoked pride, but were not instructed to imagine that they were talking to anyone in particular. After completing the task, participants rated the extent to which they felt pride on a scale of 1 (*no emotion*) to 7 (*intense emotion)* and rated how well they were able to imagine the person they were talking to on a scale of 1 (*not at all*) to 6 (*very well*).

### Measures

#### Verbal pride intensity

To examine the intensity of verbal displays of pride, three research assistants (blind to conditions) coded the videos by rating how participants described their pride-evoking accomplishments. The research assistants used a scale of 1 (*not at all*) to 5 (*very much*) to answer the following two questions: “How intense is the pride description?” and “How vivid is the event description?” The three coders’ ratings for each question were averaged for each participant and showed good interrater reliability (ICCs = .81 and .72). Due to a strong correlation between the two questions’ ratings (*r* = .65), they were averaged together to create a composite representing overall verbal pride intensity.

#### Nonverbal pride displays

The following actions were operationalized as movements indicative of pride displays: lip presses, raising of the head, head tilting up, one or both arms raised, hands on hips, arms crossed on chest, chest expanded, Duchenne smile (contraction of eye muscles and lifting of lip corners) and eye gaze directed straight ahead [3, 32–34]; the last two are less consistent characterizations of pride but were included to be thorough.

Three research assistants first identified a 3–5s segment within the recorded video that they would describe as the most meaningful moment of the behavioral task. At least two of the three coders had to agree on the 3–5s segment, which was typically the moment when participants mentioned and expressed accomplishment. Using this agreed-upon 3–5s segment as the most meaningful moment, a Facial Action Coding System-certified researcher (the second author) coded lip presses, raising of the head, and Duchenne smiles based on the occurrence of facial muscle movements [36]. Then, the same team of research assistants who coded verbal displays coded head tilting up, one or both arms raised, hands on hips, arms crossed on chest, chest expanded, and eye gaze directed straight ahead using a set of ratings, where the action was measured by their intensity level using a scale of 0 (*not at all present*) to 5 (*extreme intensity*). Coders’ intensity ratings for each expression were averaged for each participant and showed good-to-excellent interrater reliability (ICCs = .83 – .92).

### Data analyses

#### Power analyses

To be as thorough as possible, power analyses were conducted for each of three separate analyses examining (1) social context, (2) ethnicity, and (3) gender. For social context, a review of ten comparable articles on positive emotion expressions revealed a pattern of medium to large effect sizes (see S1 File for article references). Based on that review, it was estimated that a minimum of 18 participants for each of four conditions (*N* = 72) was needed for Between-Subjects Analyses of Variance (ANOVA) that had the appropriate statistical power to examine group differences with a large effect size (.40) at the recommended .80 level and an alpha level of .05 [37]. G*Power confirmed a similar sample size (*N* = 76). For ethnicity and gender, a review of appropriate articles focusing on pride and emotional experiences across cultures and genders [25, 29, 38] revealed a range of effect sizes (i.e., from small to large). Thus, power analyses for ethnicity and gender were conducted with the expectation of medium effect sizes. Based on this, it was estimated that a minimum of 52 participants for each of the three ethnicities (*N* = 156) and a minimum of 64 participants for each of the two genders (*N* = 128) were needed for Between-Subjects Analyses of Variance (ANOVAs) that had the appropriate statistical power to examine group differences with a medium effect size (.25) at the recommended .80 level and an alpha level of .05 [37]. G*Power confirmed similar sample sizes for the analyses on ethnicity (*N* = 159) and gender (*N* = 128). Thus, based on expected effect sizes and the study’s sample size, analyses on social context and gender were expected to be sufficiently powered but the analysis on ethnicity was expected to be slightly underpowered.

#### Main analytic plan

As a first step, three separate Between-Subjects Analyses of Variance (ANOVA) were conducted to determine whether there were any differences in the level of pride felt across 1) the four social context conditions, 2) the three ethnic heritage groups, and 3) women and men. Then, three separate Between-Subjects ANOVAs were conducted to examine whether the mean levels of verbal pride intensity (i.e., composite of two questions representing the intensity and vividness with which the pride experience was described) differed across (1) the four social context conditions, (2) the three ethnic heritage groups, and (3) women and men. For the nonverbal pride displays, separate Pearson Chi-Square analyses were conducted to examine whether the occurrence of lip presses, raising of the head, and Duchenne smiles (coded on a dichotomous scale of 0 or 1) differed across all groups while between-subjects ANOVAs were conducted to examine whether the mean intensity levels of the other displays—head tilting up, one or both arms raised, hands on hips, arms crossed on chest, chest expanded, and eye gaze directed straight ahead (coded on a continuous scale of 0–5)—differed across all groups. All analyses were conducted using IBM SPSS Statistics.

## Results

### Preliminary analyses

Tables 1 and 2 report the means, standard deviations, and percentages (where applicable) for verbal pride intensity and nonverbal pride displays by social context conditions, ethnic heritage groups, and gender.

**Table 1:** Mean (*SD*) values for coder ratings of verbal pride intensity by social context, ethnic heritage, and gender.

	Competitor	Loved One	Stranger	No-Context	Asian American	Latino American	European American	Women	Men
Level of Verbal Pride Intensity	2.50*(*.*64)*	2.81*(*.*85)*	2.64*(*.*60)*	2.85*(*.*62)*	2.65*(*.*72)*	2.58*(*.*57)*	3.08*(*.*73)*	2.79*(*.*65*)	2.49(.*77*)

*Note*. Verbal Pride Intensity is a composite of two questions asking about the intensity and vividness with which the pride experience was described, which were rated by research assistants on a scale of 1 (*not at all*) to 5 (*very much*).

**Table 2:** Percentages and mean (*SD*) values for nonverbal displays by social context, ethnic heritage, and gender.

	Competitor	Loved One	Stranger	No-Context	Asian American	Latino American	European American	Women	Men
Lip Presses	2.7%	2.6%	2.9%	2.9%	3.3%	2.5%	0%	3.7%	0%
Raising of Head	8.1%	5.1%	0%	2.9%	3.3%	2.5%	11.1%	2.8%	8.6%
Duchenne smiles	32.4%	41%	35.3%	31.4%	35%	35%	27.8%	38.5%	22.9%
Head Tilting Up	.81*(.93)*	.82*(1.04)*	.61*(.90)*	.77*(.77)*	.71*(.98)*	.67*(.74)*	.94*(.79)*	.77*(.96)*	.67*(.70)*
Arm/s raised	.74*(1.19)*	.53*(.79)*	.40*(.79)*	.69*(1.12)*	.71*(1.07)*	.35*(.72)*	.88*(1.27)*	.56*(.96)*	.75*(1.13)*
Hands on Hips	0*(0)*	0*(0)*	0*(0)*	0*(0)*	0*(0)*	0*(0)*	0*(0)*	0*(0)*	0*(0)*
Arms Crossed on Chest	.06*(.33)*	.08*(.44)*	.02*(.09)*	0*(0)*	.08*(.43)*	.01*(.08)*	0*(0)*	.04*(.32)*	.02*(.09)*
Chest Expanded	.22*(.47)*	.12*(.31)*	.11*(.54)*	.14*(.41)*	.08*(.25)*	.22*(.53)*	.16*(.35)*	.18*(.48)*	.06*(.25)*
Eye Gaze Straight	2.57*(1.35)*	2.1*(1.23)*	2.27*(1.22)*	2.44*(1.29)*	2.19*(1.32)*	2.32*(1.1)*	2.75*(1.41)*	2.49*(1.24)*	1.89*(1.3)*

*Note*. For Lip Presses, Raising of the Head, and Duchenne Smiles, percentage values represent those who showed that nonverbal display within each group. The rest of the nonverbal displays were rated on a scale of 0 (*not at all present*) to 5 (*extreme intensity)*.

### Experienced pride and social context

Participants reported a mean pride rating of 5.71 (*SD* = 1.51) on a scale of 1 to 7. Between-Subjects ANOVAs showed that the level of pride felt by participants during the behavioral task did not differ across the four social context conditions, *F* (3, 138) = 2.51, *p* = .06, η_p_^2^ = .05, across the three ethnic heritage groups, *F* (2, 113) = 1.34, *p* = .27, η_p_^2^ = .02, or between women and men, *F* (1, 140) < .01, *p* = .98, η_p_^2^ < .01. Participants also reported a mean rating of 3.42 (*SD* = 1.35) on a scale of 1 to 6 on how well they were able to imagine the person they were talking to in their social context condition, and this did not differ across the four social context conditions, *F* (3, 121) = 1.98, *p* = .12, η_p_^2^ = .05.

### Verbal pride intensity

**Fig 1:**
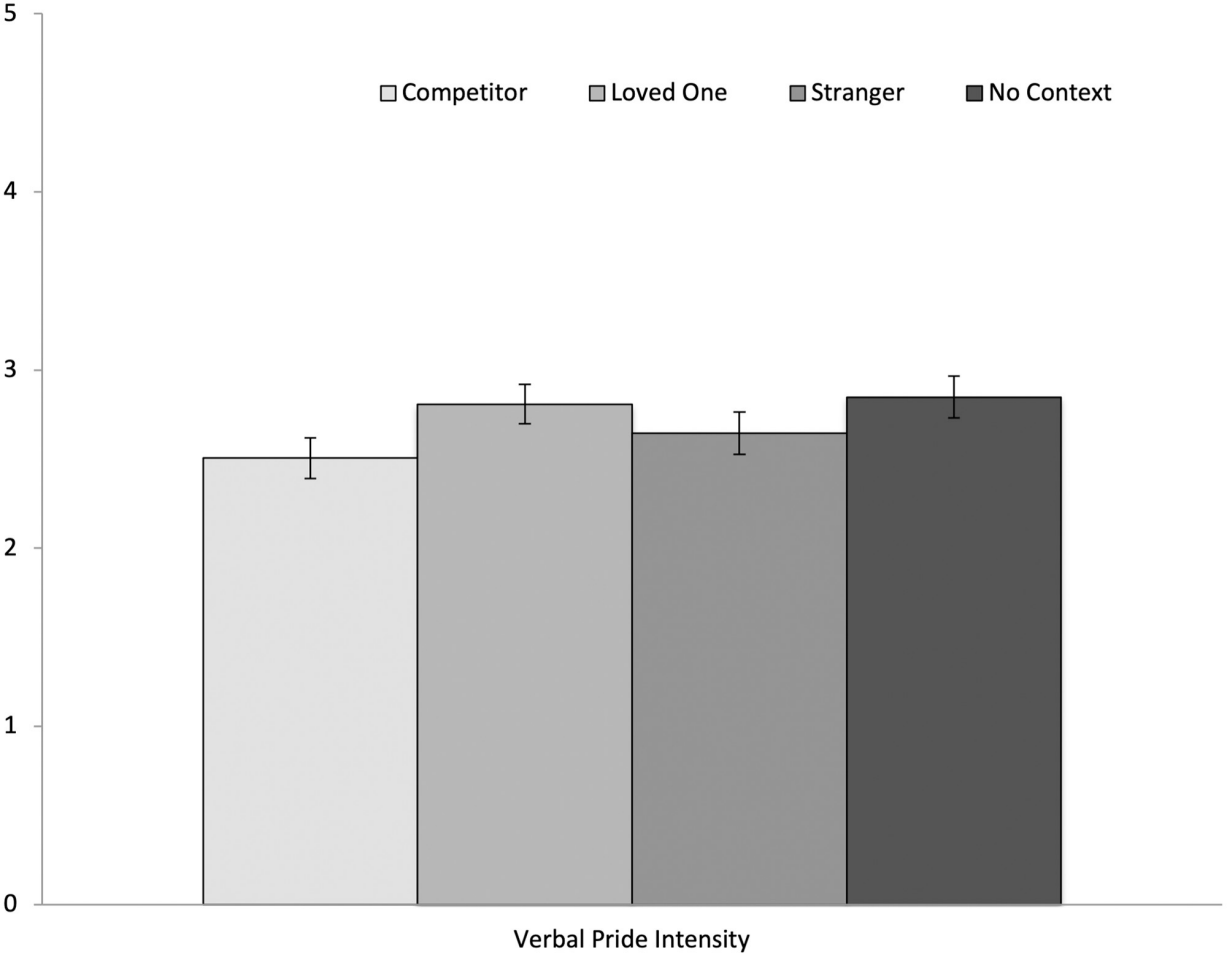
Differences in verbal pride intensity across social contexts. Error bars show the Standard Error.

**Fig 2:**
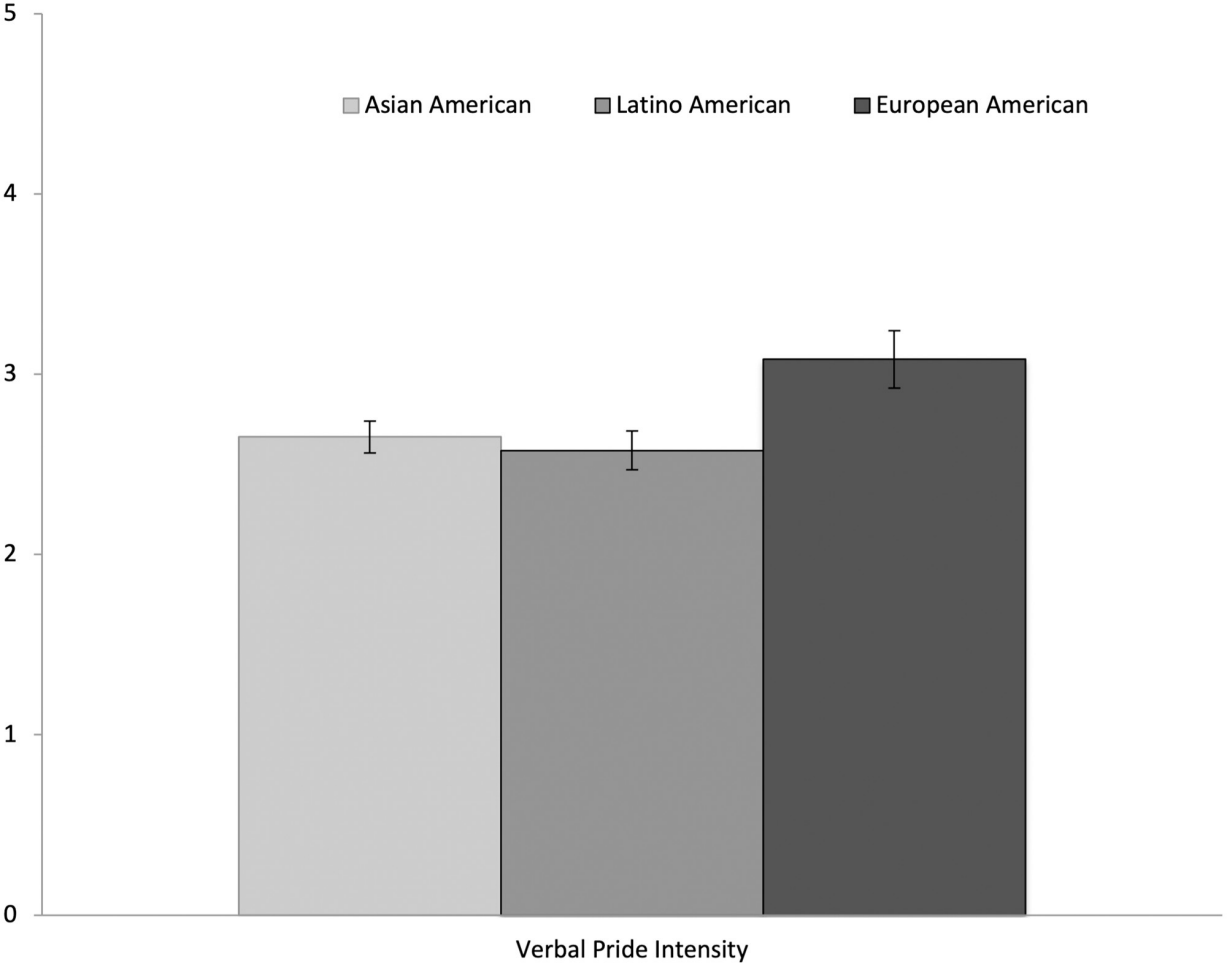
Differences in verbal pride intensity across ethnic heritage. Error bars show the Standard Error.

**Fig 3:**
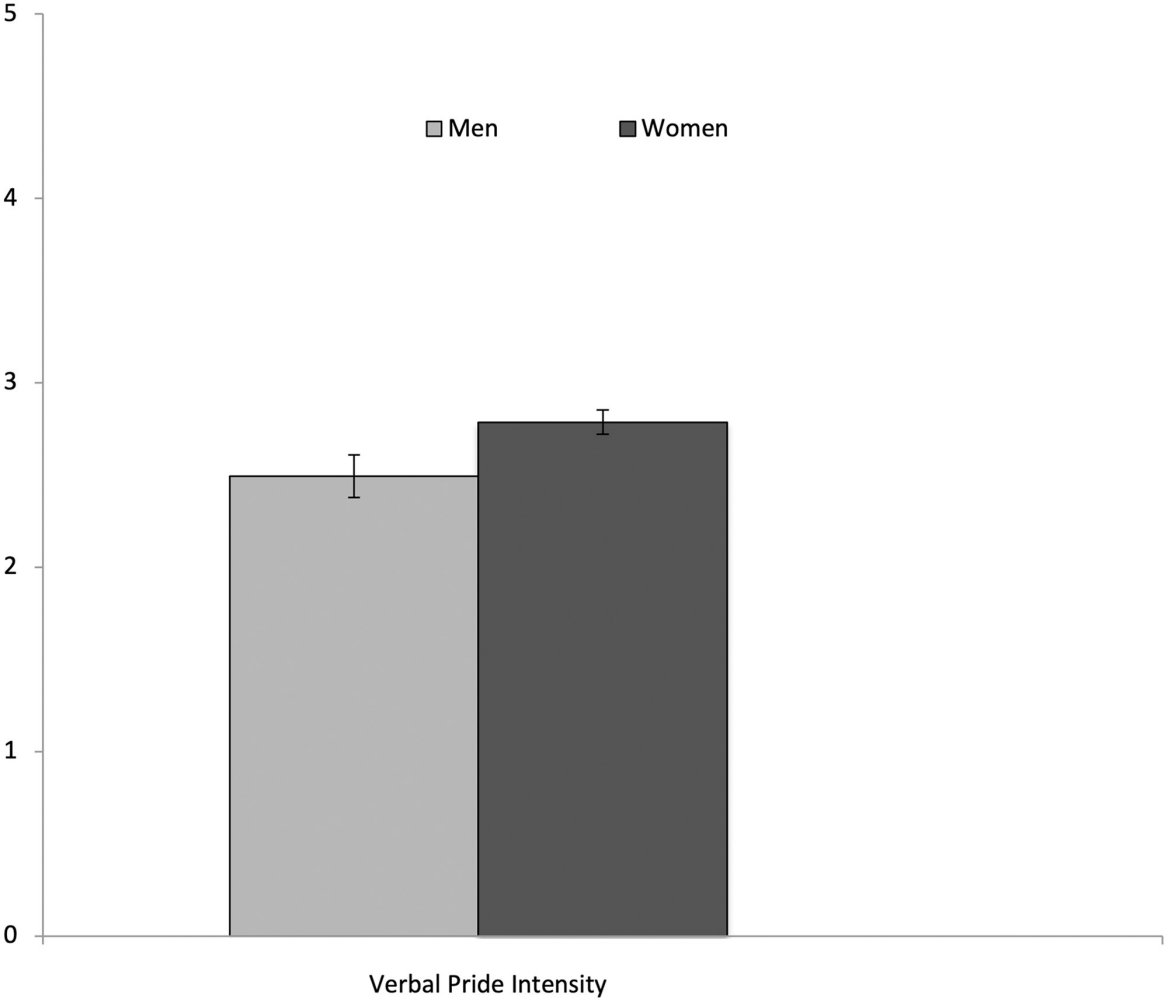
Differences in verbal pride intensity across gender. Error bars show the Standard Error.

Between-subject ANOVAs showed no differences between social context conditions in their verbal pride intensity, *F* (3, 141) = 1.91, *p* = .13., η_p_^2^ = .04 (see Fig 1). However, between-subject ANOVAs did show differences between ethnic heritage groups in their verbal pride intensity, *F* (2, 114) = 3.70, *p* = .03, η_p_^2^ = .06 (see Fig 2). Tukey post hoc tests revealed that Latino/a/x Americans showed less verbal pride intensity (*M* = 2.58; *SD* = .57) than European Americans (*M* = 3.08; *SD* = .73), *p* = .03, 95% CI (-.96, -.05), while Asian Americans (*M* = 2.65; *SD* = .72) showed marginally less verbal pride intensity than European Americans, *p* = .051, 95% CI (-.86, .001). Between-subject ANOVAs also revealed differences between women and men, such that women showed higher verbal pride intensity (*M* = 2.79; *SD* = .65) than men (*M* = 2.49; *SD* = .77), *F* (1, 141) = 4.82, *p* = .03, η_p_^2^ = .03 (see Fig 3). Exploring further, no significant interactions were found between social context, ethnicity, or gender in verbal pride intensity (*p*s > .05).

### Nonverbal pride displays

Pearson Chi-Square analyses (for presence of lip presses, raising of the head, and Duchenne smiles) and between-subject ANOVAs (for intensity levels of the other expressions) showed no differences between social context conditions (*p*s > .05) or between ethnic groups (*p*s > .05) on any nonverbal pride displays. Although there were no differences between women and men on most of the nonverbal displays, results did show differences in the intensity of the eye gaze directed straight ahead, *F* (1, 131) = 5.46, *p* = .02, η_p_^2^ = .04; women showed a more intense eye gaze (*M* = 2.49; *SD* = 1.24) than men (*M* = 1.89; *SD* = 1.31). See Table 3 for statistics.

**Table 3:** Statistics on the differences in the nonverbal displays of pride.

	Social Contexts				Ethnic Heritage				Gender			
	*χ* ^*2*^	*F*	*df*	*p*	*χ* ^*2*^	*F*	*df*	*p*	*χ* ^*2*^	*F*	*df*	*p*
Lip Presses	.01		3	1.00	.62		2	.73	1.32		1	.25
Raising of Head	3.18		3	.37	2.51		2	.28	2.25		1	.13
Duchenne smiles	.92		3	.82	.36		2	.84	2.87		1	.09
Head Tilting Up		.34	(3, 131)	.79		.56	(2, 104)	.57		.27	(1, 131)	.60
Arm/s raised		.76	(3, 131)	.52		2.08	(2, 104)	.13		.88	(1, 131)	.35
Hands on Hips		NA*	NA*	NA*		NA*	NA*	NA*		NA*	NA*	NA*
Arms Crossed on Chest		.53	(3, 131)	.66		.73	(2, 104)	.49		.26	(1, 131)	.61
Chest Expanded		.45	(3, 131)	.72		1.51	(2, 104)	.23		1.84	(1, 131)	.18
Eye Gaze Straight		.91	(3, 131)	.44		1.18	(2, 104)	.31		**5.46**	**(1, 131)**	**.02**

*Note*.

*All participants had a code of 0 (*not at all present*) for this nonverbal display.

## Discussion

The current study sought to better understand the potentially motivated management of pride by examining whether the display of pride, both in terms of its verbal intensity and its nonverbal display, would vary by social contexts, ethnic heritage groups, or gender. Results showed that the display of pride did not differ by social context but ethnic heritage and gender variation were observed. Specifically, Latino/a/x Americans and Asian Americans showed less verbal pride intensity than European Americans, although only the difference between Latino/a/x Americans and European Americans reached statistical significance. These ethnicity findings are novel, advance the study of pride display, and have implications for better understanding interpersonal emotion regulation in diverse settings. It was also found that women showed higher verbal pride intensity than men, an unexpected finding that reveals nuance in the ways people manage this complex emotion. Together, the findings of the study suggest that culture and gender are relevant for understanding the display of pride, and this variation may be most salient in verbal aspects of the display.

### Social context

Findings showed no observed differences in the level of pride felt across conditions. Participants in all four of the experimental social contexts reported comparable pride experiences. Pride experiences did not differ in their verbal intensity whether described to competitors, loved ones, strangers, or in the no-context condition. This differs from other findings on pride display variation across social contexts that manipulated both imagined achievements and, similar to this study, imagined social contexts [11]. As this study’s findings differ, there needs to be more research on factors that might lead pride displays to vary, including the possibility of co-variation with other positive emotions (e.g., joy or gratitude). If there is indeed co-variation with other positive emotions, the overall experience may be perceived to have positive consequences for social relationships, and this may reduce the likelihood that individuals want to inhibit their pride displays. On the other hand, people may be exclusively focused on their positive feelings, and only later become attuned to the potential negative social consequences that would have otherwise led them to inhibit their pride displays.

There were no observed differences in nonverbal displays. This may have been due to a floor effect (i.e., lack of base rates) in these behaviors or general management in not displaying them. For example, only four participants showed lip presses and only six showed raising of the head. As this differs from prototypical pride expressions [32, 34], further investigation may be needed to better understand spontaneous pride displays across situations, as well as examine the conditions under which individuals may broadly inhibit pride displays.

### Ethnicity

Ethnic group differences were observed in verbal pride intensity, but not in nonverbal pride displays. It was expected that Asian Americans would display less pride verbally because of research suggesting that pride may be more fraught in this specific collectivistic context. Prior research has found less perceived pride displayed and experienced by individuals of Asian heritage compared to individuals of European American heritage [23] and individuals of Latino/a/x heritage [21]. However, this study’s findings instead suggest more similarity between Asian Americans with European Americans and Latino/a/x Americans. Asian Americans only marginally differed in verbal pride intensity from European Americans and no differences between Asian Americans and Latino/a/x Americans were observed. While the social norms of the two collectivistic cultures (i.e., Asian Americans and Latino/a/x Americans) vary, both may be similarly motivated when it comes to managing pride experience.

Felt pride did not vary but results did find that Latino/a/x Americans described the pride experience less intensely than European Americans. Although research has reported differences in pride between Latino/a/x Americans and European Americans in the opposite direction [21], the study’s finding broadly aligns with literature that describes collectivistic cultures as prioritizing the group over the individual [39] and as considering pride to be more undesirable [22]. However, as the samples in these and other similar findings are usually East Asian, not Latino/a/x, the current findings cannot be completely explained by the literature that has highlighted differences between individualistic and collectivistic cultures. Thus, further investigation will be needed, particularly in academic contexts, as research shows that Latino/a/x college students report higher family achievement guilt (i.e., guilt over surpassing the achievements of the family) than European Americans [40]. More research on this topic may help shed novel insight on the complicated experience of achievement in academic settings for ethnic minority students; in this setting Latino/a/x Americans may be more attuned to the potentially negative consequences of verbally displaying pride compared to European Americans. Overall, the patterns observed in this study highlight the need to reach a better understanding of the nuances in the ways that the three ethnic heritage groups studied here manage pride displays.

### Gender

Research suggests that maleness is associated with pride, as men may have heightened attunement to status and seek it more relative to women [30], but we found that women described their pride experience more intensely, even when feeling a similar level of pride than men. This aligns with past literature that has shown that while women experience emotion similarly to men, they are more expressive [41], especially with positive emotion [42]. The women in the study sample also did not inhibit their nonverbal displays or their eye gazes as they described their prideful accomplishments in comparison to men. Here again, the patterns observed in this study highlight that, in this case, the relation of gender with pride displays is more nuanced than research has thus far uncovered. This fascinating possibility merits additional research that could further advance the study of gendered patterns of interpersonal emotion regulation.

### Study strengths

This study had several strengths. First, the study examined individuals whose ethnic heritage backgrounds are linked to two different forms of collectivistic culture and whose varying ways of managing pride have scarcely been studied. Literature on pride and positive emotion has focused mostly on Asian cultures as representative of collectivistic cultures, not Latino cultures, despite evidence increasingly indicating East Asian-Latin American variation in emotion [21, 25]. Thus, the study’s examination of variation within collectivistic cultures is especially important, and it will be essential going forward for researchers to examine and distinguish between the two to continue building a greater understanding of the variation possible within the broader collectivistic framework. Another strength was that the study examined how pride is expressed in two different ways, through verbal intensity and nonverbal displays. This allowed the study to capture more nuance in the ways that people may be motivated to show their pride. An additional strength was that an experimental design—a behavioral task—was employed that allowed observation of participants as they verbally described a pride achievement. Other strengths include using a stranger condition, which van Osch and colleagues (2019) acknowledged might influence findings, and not limiting the length of time participants could use to describe their prideful experiences.

### Study limitations and recommendations

There were also limitations in the current study. Although the experimental design was influenced by previous studies [8, 11, 23] and involved participants describing a real prideful accomplishment, participants imagined the social context condition, and the study’s manipulation check revealed that the task was only moderately successful in leading participants to imagine their condition. What this suggests is that the task might reflect people’s ideas about pride more than an experience of pride. If so, useful information has still been provided about how people of varying ethnic groups and genders think pride should be expressed. Another possible limitation was that the study’s samples of Asian Americans and Latino/a/x Americans may not have been the best representatives for examining cultural variation, as most participants were born in the United States and all came from American universities. Future studies will do well to examine pride display differences across people from different cultures who do reside in the countries their heritage originates from and are immersed in the country’s languages and norms (e.g., Latino/a/x individuals in Latin America). Additionally, although a priori power analyses indicated that the sample size was sufficient to detect large and medium effects for social context and gender differences, G*Power post hoc power analyses revealed that considering the findings showing small-to-medium effect sizes (η_p_^2^ = .03 – .06), both ethnic and gender differences, along with the lack of social context differences, were underpowered.

Despite the limitations, the study’s findings encourage further examination of pride displays in diverse settings. For example, future work may deepen understanding of the conditions under which women engage in status-signaling. Studies of people who identify as nonbinary will be particularly insightful; societal understanding of gender is changing and it will be important to consider how social norms around status-seeking change too. Future studies should also examine how pride is displayed across different status hierarchies. For example, status-shifting individuals who have experienced upward social mobility may be learning new ways to manage status-focused complex emotions. Also integral to the overall study of pride will be research that examines pride displays in interpersonal, dyadic settings. Research focusing on the receiving end of pride displays, together with the delivery of the display, will be crucial to better understanding the complex interpersonal dynamics that may occur with pride.

## Conclusion

Pride is a unique emotion. While people recognize the experience of pride and what its behavioral display looks like, displaying pride can have negative social consequences. For this reason, the current study aimed to examine whether people manage its display differently, both verbally and nonverbally. This study adds new evidence that advances the current understanding of pride by suggesting less social context variation and more human diversity variation (i.e., ethnicity and gender). Latino/a/x Americans verbally display it less intensely than European Americans while women verbally display it more intensely than men. Overall, the study’s findings support the view that the experience and expression of pride are consistent across contexts, but people may manage their pride experiences differently and these nuances are highly worthy of additional study for understanding pride specifically and interpersonal emotion regulation broadly.

## Supporting information

S1 File (DOCX)

## References

[1] Gable SL, Reis HT, Impett EA, Asher ER. What do you do when things go right? The intrapersonal and interpersonal benefits of sharing positive events. J Pers Soc Psychol. 2004;87(2):228–45. doi: 10.1037/0022-3514.87.2.228 15301629

[2] Darwin C. The expression of the emotions in man and animals. London: John Murray; 1872.

[3] Tracy JL, Robins RW. Show your pride: Evidence for a discrete emotion expression. Psychol Sci. 2004 Mar;15(3):194–7. doi: 10.1111/j.0956-7976.2004.01503008.x 15016291

[4] Tracy JL, Robins RW. The nonverbal expression of pride: Evidence for cross-cultural recognition. J Pers Soc Psychol. 2008;94(3):516–30. doi: 10.1037/0022-3514.94.3.516 18284295

[5] Gable SL, Reis HT. Good news! Capitalizing on positive events in an interpersonal context. In: Advances in Experimental Social Psychology [Internet]. Elsevier; 2010 [cited 2021 Nov 10]. p. 195–257. https://linkinghub.elsevier.com/retrieve/pii/S0065260110420043

[6] Keltner D, Kring AM. Emotion, social function, and psychopathology. Rev Gen Psychol. 1998 Sep;2(3):320–42.

[7] Tracy JL, Robins RW. The psychological structure of pride: A tale of two facets. J Pers Soc Psychol. 2007;92(3):506–25. doi: 10.1037/0022-3514.92.3.506 17352606

[8] Kalokerinos EK, Greenaway KH, Pedder DJ, Margetts EA. Don’t grin when you win: The social costs of positive emotion expression in performance situations. Emotion. 2014;14(1):180–6. doi: 10.1037/a0034442 24188058

[9] Lange J, Crusius J. The tango of two deadly sins: The social-functional relation of envy and pride. J Pers Soc Psychol. 2015;109(3):453–72. doi: 10.1037/pspi0000026 26167795

[10] Tesser A. Toward a self-evaluation maintenance model of social behavior. In: Berkowitz L, editor. Advances in experimental social psychology, Vol 21: Social psychological studies of the self: Perspectives and programs [Internet]. Academic Press (San Diego, CA, US); 1988 [cited 2021 Nov 10]. p. 181–227, Chapter x, 361 Pages. https://www.proquest.com/psycinfo/docview/617497051/F9454F91C2B740F0PQ/2

[11] van Osch Y, Zeelenberg M, Breugelmans SM, Brandt MJ. Show or hide pride? Selective inhibition of pride expressions as a function of relevance of achievement domain. Emotion. 2019 Mar;19(2):334–47. doi: 10.1037/emo0000437 29878803

[12] Chung JM, Robins RW. Exploring cultural differences in the recognition of the self-conscious emotions. Bard KA, editor. PLOS ONE. 2015 Aug 26;10(8):27. doi: 10.1371/journal.pone.0136411 26309215 PMC4550404

[13] Shi Y, Chung JM, Cheng JT, Tracy JL, Robins RW, Chen X, et al. Cross-cultural evidence for the two-facet structure of pride. J Res Personal. 2015 Apr;55:61–74. doi: 10.1016/j.jrp.2015.01.004 27158171 PMC4856297

[14] Tracy JL, Matsumoto D. The spontaneous expression of pride and shame: Evidence for biologically innate nonverbal displays. Proc Natl Acad Sci. 2008 Aug 19;105(33):11655–60. doi: 10.1073/pnas.0802686105 18695237 PMC2575323

[15] Durkee PK, Lukaszewski AW, Buss DM. Pride and shame: Key components of a culturally universal status management system. Evol Hum Behav. 2019 Sep;40(5):470–8.

[16] Sznycer D, Al-Shawaf L, Bereby-Meyer Y, Curry OS, De Smet D, Ermer E, et al. Cross-cultural regularities in the cognitive architecture of pride. Proc Natl Acad Sci. 2017 Feb 21;114(8):1874–9. doi: 10.1073/pnas.1614389114 28167752 PMC5338381

[17] Sznycer D, Xygalatas D, Alami S, An XF, Ananyeva KI, Fukushima S, et al. Invariances in the architecture of pride across small-scale societies. Proc Natl Acad Sci. 2018 Aug 14;115(33):8322–7. doi: 10.1073/pnas.1808418115 30068602 PMC6099881

[18] Sznycer D. Forms and functions of the self-conscious emotions. Trends Cogn Sci. 2019 Feb;23(2):143–57. doi: 10.1016/j.tics.2018.11.007 30583948

[19] Senft N, Campos B, Shiota MN, Chentsova-Dutton YE. Who emphasizes positivity? An exploration of emotion values in people of Latino, Asian, and European heritage living in the United States. Emotion. 2021 Jun;21(4):707–19. doi: 10.1037/emo0000737 32191097

[20] Tsai JL. Ideal affect: Cultural causes and behavioral consequences. Perspect Psychol Sci. 2007 Sep;2(3):242–59. doi: 10.1111/j.1745-6916.2007.00043.x 26151968

[21] Scollon CN, Diener E, Oishi S, Biswas-Diener R. Emotions across cultures and methods. J Cross-Cult Psychol. 2004 May;35(3):304–26.

[22] Eid M, Diener E. Norms for experiencing emotions in different cultures: Inter- and intranational differences. J Pers Soc Psychol. 2001;81(5):869–85. doi: 10.1037//0022-3514.81.5.869 11708563

[23] van Osch Y, Zeelenberg M, Breugelmans SM. On the context dependence of emotion displays: Perceptions of gold medalists’ expressions of pride. Cogn Emot. 2016 Oct 2;30(7):1332–43. doi: 10.1080/02699931.2015.1063480 26208599

[24] Corona K, Senft N, Campos B, Chen C, Shiota M, Chentsova-Dutton Y. Ethnic variation in gratitude and well-being. Emotion. 2020 Apr;20(3):518–24. doi: 10.1037/emo0000582 30869943

[25] Ruby MB, Falk CF, Heine SJ, Villa C, Silberstein O. Not all collectivisms are equal: Opposing preferences for ideal affect between East Asians and Mexicans. Emotion. 2012;12(6):1206–9. doi: 10.1037/a0029118 22775131

[26] Campos B, Kim HS. Incorporating the cultural diversity of family and close relationships into the study of health. Am Psychol. 2017 Sep;72(6):543–54. doi: 10.1037/amp0000122 28880101

[27] The authors use the term “Hispanic” when citing previous research because that is how the samples were identified by researchers; however, the term “Latino” is used to refer to the culture more broadly and “Latino/a/x” to refer to the specific individuals in the current study. These terms are not necessarily synonymous, but all refer to the same population of Latin American individuals living in the United States. These decisions reflect the authors’ effort to be considerate of the multiple viewpoints on preferred terms among this in-group.

[28] Acevedo AM, Herrera C, Shenhav S, Yim IS, Campos B. Measurement of a Latino cultural value: The Simpatía scale. Cultur Divers Ethnic Minor Psychol. 2020 Oct;26(4):419–25. 32105107 10.1037/cdp0000324

[29] Else-Quest NM, Higgins A, Allison C, Morton LC. Gender differences in self-conscious emotional experience: A meta-analysis. Psychol Bull. 2012 Sep;138(5):947–8. doi: 10.1037/a0027930 22468881

[30] Schram A, Brandts J, Gërxhani K. Social-status ranking: A hidden channel to gender inequality under competition. Exp Econ. 2019 Jun 15;22(2):396–418.

[31] Smith JS, LaFrance M, Knol KH, Tellinghuisen DJ, Moes P. Surprising smiles and unanticipated frowns: How emotion and status influence gender categorization. J Nonverbal Behav. 2015 Jun;39(2):115–30.

[32] Campos B, Shiota MN, Keltner D, Gonzaga GC, Goetz JL. What is shared, what is different? Core relational themes and expressive displays of eight positive emotions. Cogn Emot. 2013 Jan;27(1):37–52. doi: 10.1080/02699931.2012.683852 22716231

[33] Ekman P, Davidson RJ, Friesen WV. The Duchenne smile: Emotional expression and brain physiology: II. J Pers Soc Psychol. 1990 Feb;58(2):342–53. 2319446

[34] Tracy JL, Robins RW. The prototypical pride expression: Development of a nonverbal behavior coding system. Emotion. 2007;7(4):789–801. doi: 10.1037/1528-3542.7.4.789 18039048

[35] 26 participants were excluded because they self-identified as “other” and two were excluded due to missing data.

[36] Ekman P. F W. Facial Action Coding System: A technique for the measurement of facial movement. Palo Alto (CA): Consulting Psychologists Press.; 1978.

[37] Cohen J. A power primer. Psychol Bull. 1992;112(1):155–9. doi: 10.1037//0033-2909.112.1.155 19565683

[38] Webb L, Stegall S, Mirabile S, Zeman J, Shields A, Perry-Parrish C. The management and expression of pride: Age and gender effects across adolescence. J Adolesc. 2016 Oct;52:1–11. doi: 10.1016/j.adolescence.2016.06.009 27454777

[39] Carducci BJ. Expressions of the self in individualistic vs. collective cultures: A cross-cultural-perspective teaching module. Psychol Learn Teach. 2012 Sep;11(3):413–7.

[40] Covarrubias R, Fryberg SA. Movin’ on up (to college): First-generation college students’ experiences with family achievement guilt. Cultur Divers Ethnic Minor Psychol. 2015;21(3):420–9. doi: 10.1037/a0037844 25198416

[41] Kring AM, Gordon AH. Sex differences in emotion: Expression, experience, and physiology. J Pers Soc Psychol. 1998 Mar;74(3):686–703. doi: 10.1037//0022-3514.74.3.686 9523412

[42] LaFrance M, Hecht MA, Paluck EL. The contingent smile: A meta-analysis of sex differences in smiling. Psychol Bull. 2003;129(2):305–34. doi: 10.1037/0033-2909.129.2.305 12696842

