# Correction: Displaying pride: Variation by social context, ethnic heritage, and gender?

**DOI:** 10.1371/journal.pone.0315048

**Published:** 2024-12-02

**Authors:** The PLOS ONE Staff

The first author’s initials appear incorrectly in the citation. The correct citation is: Sanchez Hernandez H, Hovasapian A, Campos B (2023) Displaying pride: Variation by social context, ethnic heritage, and gender? PLOS ONE 18(4): e0285152. https://doi.org/10.1371/journal.pone.0285152.

In the Verbal pride intensity subsection of the Results, there is an error in the first sentence of the paragraph. The correct sentence is: Between-subject ANOVAs showed no differences between social context conditions in their verbal pride intensity, *F* (3, 141) = 1.91, p = .13, ηp2 = .04 (see Fig 1).

In the Study limitations and recommendations subsection of the Discussion, there is an error in the fifth sentence of the first paragraph. The correct sentence is: Another possible limitation was that the study’s samples of Asian Americans and Latino/a/x Americans may not have been the best representatives for examining cultural variation, as most participants were born in the United States and all came from an American university.

The publisher apologizes for the errors.
