# Supplementary material for: Displaying pride: Variation by social context, ethnic heritage, and gender?
Source: PLoS One. 2023 Apr 28;18(4):e0285152. doi: 10.1371/journal.pone.0285152 (PMC10146430; doi:10.1371/journal.pone.0285152)
Supplement: S1 File — (DOCX) [file pone.0285152.s001.docx]

**Power Analysis References**

Chung, J. M., & Robins, R. W. (2015). Exploring cultural differences in the recognition of the self-conscious emotions*. PLoS One, 10*(8) <http://dx.doi.org/10.1371/journal.pone.0136411>

Furley, P., Moll, T., & Memmert, D. (2015). “Put your hands up in the air”? The interpersonal effects of pride and shame expressions on opponents and teammates.*Frontiers in Psychology, 6*, 20. Retrieved October 8, 2019, from <https://search.proquest.com/docview/1792778518?accountid=14509>

Kalokerinos, E. K., Greenaway, K. H., Pedder, D. J., & Margetts, E. A. (2014). Don’t grin when you win: The social costs of positive emotion expression in performance situations. *Emotion, 14*(1), 180-186. <http://dx.doi.org/10.1037/a0034442>

Shariff, A. F., Tracy, J. L., & Markusoff, J. L. (2012). (Implicitly) judging a book by its cover: The power of pride and shame expressions in shaping judgments of social status.*Personality and Social Psychology Bulletin, 38*(9), 1178-1193. <http://dx.doi.org/10.1177/0146167212446834>

Tracy, J. L., & Robins, R. W. (2007a). The prototypical pride expression: Development of a nonverbal behavior coding system. *Emotion, 7*(4), 789–801. <http://dx.doi.org/10.1037/1528-3542.7.4.789>

Tracy, J. L., & Robins, R. W. (2007b). The psychological structure of pride: A tale of two facets. *Journal of Personality and Social Psychology, 92*(3), 506-525 <http://dx.doi.org/10.1037/0022-3514.92.3.506>

van Osch, Y., Zeelenberg, M., & Breugelmans, S. M. (2016). On the context dependence of emotion displays: Perceptions of gold medalists’ expressions of pride. *Cognition and Emotion, 30*(7), 1332-1343. <http://dx.doi.org/10.1080/02699931.2015.1063480>

van Osch, Y., Zeelenberg, M., Breugelmans, S. M., & Brandt, M. J. (2019). Show or hide pride? Selective inhibition of pride expressions as a function of relevance of achievement domain.*Emotion, 19*(2), 334-347. <http://dx.doi.org/10.1037/emo0000437>

Webb, L., Stegall, S., Mirabile, S., Zeman, J., Shields, A., & Perry-Parrish, C. (2016). The management and expression of pride: Age and gender effects across adolescence.*Journal of Adolescence, 52*, 1-11. <http://dx.doi.org/10.1016/j.adolescence.2016.06.009>

Wubben, M. J. J., De Cremer, D., & van Dijk, E. (2012). Is pride a prosocial emotion? interpersonal effects of authentic and hubristic pride.*Cognition and Emotion, 26*(6), 1084-1097. <http://dx.doi.org/10.1080/02699931.2011.646956>
